# A Case of Allergic Bronchopulmonary Aspergillosis With Bronchial Asthma

**DOI:** 10.7759/cureus.29552

**Published:** 2022-09-25

**Authors:** Pratik Bharat Rajpopat, Brinda Niravkumar Desai, Sheeraz Abro, Tulika Garg, Oyovwike S Amedu, Toluwalope F Ejiyooye, Ayesha Fonseca, Taha Sajjad, Eloho Hambolu, Aadil Khan

**Affiliations:** 1 Internal Medicine, Gujarat Medical Education & Research Society (GMERS) Medical College and Hospital, Valsad, IND; 2 Internal Medicine, Chandka Medical College, Larkana, PAK; 3 Internal Medicine, Government Medical College & Hospital, Chandigarh, IND; 4 Medicine, Federal Medical Centre Abeokuta, Abeokuta, NGA; 5 Family Medicine, Brooke Army Medical Center, San Antonio, USA; 6 Medicine, American University of Integrative Sciences (AUIS) School of Medicine, Barbados, BRB; 7 Medical Education, Mountain Vista Medical Center (MVMC), Phoenix, USA; 8 Family Medicine, Sacred Heart Hospital, Abeokuta, NGA; 9 Internal Medicine, Lala Lajpat Rai (LLR) Hospital, Kanpur, IND

**Keywords:** abpa, type 1 hypersensitivity, eosinophila, asthma, allergic broncho-pulmonary aspergillosis

## Abstract

Allergic bronchopulmonary aspergillosis (ABPA) is a fungal hypersensitivity reaction in chronic lung diseases like bronchial asthma and cystic fibrosis. It is caused by an allergic reaction to aspergillus antigen in the lung mucus resulting in airway inflammation and damage. This condition usually presents in a patient with asthma as a poorly controlled disease with recurrent infection symptoms that do not respond to standard antibiotic therapy. Diagnosis is made by chest X-ray, computed tomography, eosinophilia, and raised serum IgE on serology and immunological tests for aspergillus antigen. Lack of diagnosis and treatment of the condition can lead to respiratory failure from bronchiectasis and pulmonary fibrosis.

## Introduction

Aspergillus is a ubiquitous fungus known to cause various allergic pulmonary diseases like allergic bronchopulmonary aspergillosis (ABPA), allergic Aspergillus sinusitis, IgE-mediated asthma, and hypersensitivity pneumonitis [1]. ABPA is caused by an inflammatory response to Aspergillus in the lung mucus of people with predisposing lung diseases like persistent asthma and cystic fibrosis. Several immune factors like atopy and some immunogenic human leukocyte antigen (HLA) phenotypes in asthmatic patients, and genetic factors like the cystic fibrosis transmembrane conductance regulator (CFTR) gene mutation in patients with cystic fibrosis, lead to an increased risk of ABPA in these patients [2,3]. This occurs due to the colonization of the fungus in the airway and progressive lung inflammation, causing bronchospasm and mucus production manifested as coughing, difficulty breathing, and obstruction of the airways. Long-term sequelae of this disease due to delay in diagnosis and undertreatment can result in worsening lung function owing to pulmonary fibrosis, bronchiectasis with chronic mucus production, and increasingly severe persistent asthma with loss of lung function [4].

ABPA occurs in 1%-2% of patients with asthma and 1%-7.8% of patients with cystic fibrosis [5]. However, the prevalence of ABPA among asthmatics has been difficult to establish because of the lack of standard diagnostic criteria. It should be suspected in a patient with chronic airway limitation associated with a predisposing lung disease like asthma or cystic fibrosis. Diagnostic workup for ABPA includes a positive skin test for Aspergillus, elevated serum IgE level (>416 IU/L), fungal-specific IgG and IgE antibodies, and imaging modalities [6].

## Case presentation

A 44-year-old male presented with chief complaints of shortness of breath and cough with expectoration for the last four weeks. His cough was productive, yellowish with no blood-stained. He also complained of mild intermittent fever for the last month and was relieved by taking paracetamol. He had no history of travel, tuberculosis, alcohol, or illicit drug use. He was diagnosed with bronchial asthma at the age of 26 years and gradually progressed over the past ten years. His symptoms were mild and controlled with a short-acting beta-agonist and a moderate dose of inhaled corticosteroid. He experienced exertional wheezing one year ago, and his forced expiratory volume in 1 second (FEV1)/forced vital capacity (FVC) ratio was 64%, and peak expiratory flow of 3.37 L/s. He was managed with a long-acting beta-agonist (LABA), inhaled corticosteroids (ICS), leukotriene receptor antagonist (LRTA), and long-acting muscarinic antagonist (LAMA) and discharged when his condition was stabilized.

**Figure 1:**
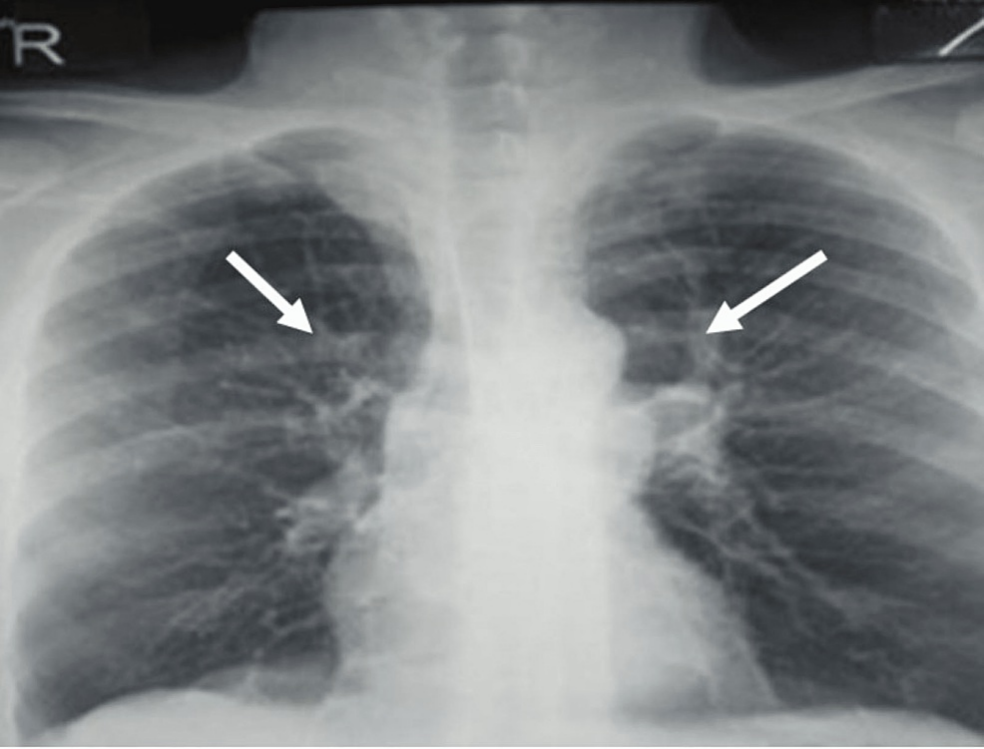
Chest X-ray showing linear opacity associated with retraction signs in the hilar regions.

On examination, he was febrile (100°F), with a respiratory rate of 25/minute, heart rate of 92/minute, blood pressure of 130/85 mmHg, and oxygen saturation of 90% at room air. He had expiratory wheezing in both lobes with high-pitched conducting sounds on auscultation. The rest of the physical examination was unremarkable. Patients' hematological and biochemical parameters are shown in Table 1. His recent chest X-ray revealed opacities in both lungs, most likely central bronchiectasis, as shown in Figure 1.

**Table 1:** The results of initial laboratory tests. LDH: lactate dehydrogenase, AST: aspartate aminotransferase, RBC: red blood cell count.

Parameter	Lab value (Reference range)
Hemoglobin	11.2 (12-16.5) g/dl
Mean corpuscular hemoglobin	33 (27-32) pg
Mean corpuscular volume	83 (80-100) fL
RBC	4.1 (4.2-5.4) million cells/µL
White cell count	13100 (4000-11000)/µL
Neutrophils	41 (40-70)%
Monocytes	3 (2-10)%
Lymphocytes	19 (20-50)%
Eosinophils	18 (01-06)%
Platelet count	230,000 (150,000-450,000)/µL
LDH	297 (105-333) IU/L
Erythrocyte sedimentation rate	21 (1-13) mm/hr
Alanine aminotransferase	45 (<40) IU/L
AST	51 (<35) IU/L
Alkaline phosphatase	202 (<240) IU/L
D-dimer	349 (220-500 ng/ml)

**Figure 2:**
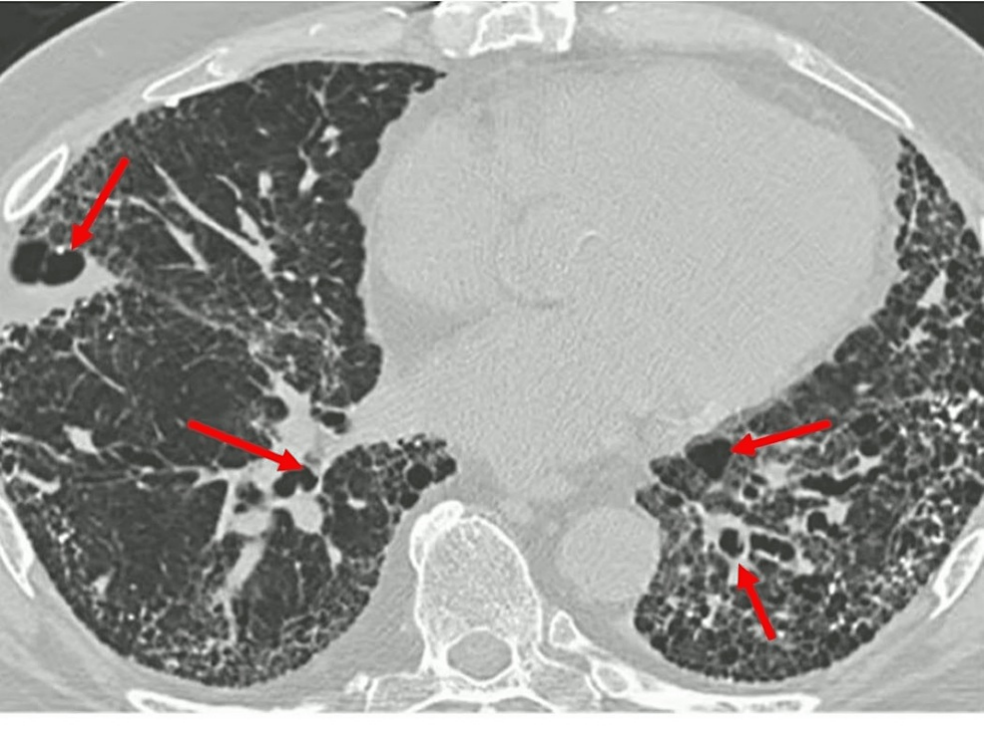
High-resolution CT chest demonstrating ground glass opacities, airway dilation, and thickened airways manifesting bronchiectasis. CT: computed tomography

A high-resolution computed tomography (HRCT) of the chest was performed, which revealed ground glass opacities (GGOs), airway dilation, and thickness in both lungs with minimal focal distension of the airways (Figure 2). A provisional diagnosis of pneumonia secondary to the predisposition of microbiological infection was made. However, the patient did not respond well to intravenous antibiotic treatment, high-dose steroids, LABA, LAMA, and theophylline.

Later, it was identified that patient had type I aspergillus skin test positive in addition to elevated total IgE levels of more than 2700 IU/mL. Moreover, the blood workup showed the presence of peripheral eosinophilia. His serum was positive for Aspergillus-specific Ige (7.1 UA/ml). Blood culture did not grow any organism, sputum culture was negative for A. fumigatus, and KOH stain was not performed. He underwent bronchoscopy, and bronchial washing revealed eosinophilia, and bronchial fluid did not grow any organism. He was diagnosed with ABPA based on the history of bronchial asthma, elevated total IgE levels, and presence of precipitating antibodies and A. fumigatus-specific elevated IgE antibodies. He was managed with systemic prednisone 30mg daily, LAABA, and itraconazole 200mg daily. His condition started improving with a reduction of eosinophilia and total IgE levels. Systemic steroids were tapered and discontinued over four weeks, aside from a short course of steroids for asthma exacerbation. He was evaluated every two months. Six months later, there was a significant clinical improvement, and he was compliant with his daily activities.

## Discussion

Aspergillus fumigatus (A. fumigatus) hypersensitivity is the cause of the respiratory condition known as ABPA [1]. It is a type 2 hypersensitive lung disease characterized by bronchial obstruction, bronchiectasis, pulmonary and peripheral eosinophilia, recurring temporary chest radiography infiltrates, especially after an exacerbation of asthma, and bronchial colonization with A. fumigatus. Following Aspergillus conidia colonization of the bronchi in susceptible people, illness develops. The fungal hyphae spread, and allergens are released, resulting in a chronic airway inflammation that produces excessive sticky mucus and impairs mucociliary function. Poorly managed asthma, bronchiectasis, and recurrent pulmonary infiltrates, which in some instances might result in lung fibrosis, are the clinical characteristics of ABPA [2].

It is hypothesized that the increased frequency and activity of A. fumigatus-specific Th2 CD4+ cells contribute to the development of ABPA in genetically vulnerable patients [3]. The prevalence of ABPA is thought to be between 1-2% in adult asthma patients and between 2-15% in cystic fibrosis patients. Drug toxicity is frequently observed during therapeutic therapy, with severe uncontrolled asthma being the most common association with ABPA. Asthma is characterized by the airways' hypersensitivity to outside stimuli. There are now a lot of studies to determine how environmental and genetic factors affect the severity of asthma and how it develops. There has been a significant increase in evidence connecting fungus to asthma in recent years. Some vital clinical conditions associated with fungal sensitization and hypersensitive immune response include allergic ABPA, severe asthma with fungal sensitization (SAFS), and allergic fungal rhinosinusitis (AFRS). Although a relationship with several different fungi has been identified, Aspergillus is the most frequently implicated in these diseases. Patients with asthma may become IgE-sensitive to several molds, including A. fumigatus. Since ABPA's symptoms may be extreme and result in pulmonary fibrosis, it is essential to detect ABPA in asthma patients who do not respond well to conventional treatment. This is because ABPA might negatively influence the quality of life without proper management.

In people with asthma, ABPA is an uncommon but serious illness that causes significant morbidity. The lack of specificity in clinical signs makes early identification challenging. Although it has been known for decades, the connection between severe asthma and fungi is still not fully understood. Recent research has identified a novel phenotype of asthma called severe SAFS, which represents a progression from allergic asthma to SAFS and then to ABPA [4]. In India, fungi are among the most frequent infections contributing to severe allergic asthma. All people are exposed to fungi, although most people do not become sensitized to them. Yeasts, the Aspergillus and Penicillium genera, and other fungi that can thrive at body temperature can infect the lungs and cause pulmonary diseases. Aspergillus fumigatus is the fungus that affects the lungs the most frequently, while other fungi can also infect the lungs [5]. Recombinant A. fumigatus antigens induced IgE serological responses to several antigens in ABPA and Aspergillus-allergic asthmatic patients. Although its pathogenetic importance is unclear, this offers significant diagnostic value in identifying the specific asthma phenotype [6].

Early identification and management of ABPA can lessen adverse effects and fibrosis-related irreparable lung damage. One method of treating ABPA is by lowering acute inflammation. A dual treatment strategy is necessary. Corticosteroids and antifungal azoles can lessen the activation of the antigenic response [7]. There have been two modest, short-term, double-blind, randomized, placebo-controlled trials for asthmatic ABPA and none for cystic fibrosis ABPA. Alternative methods of antifungal therapy exist that do not have systemic side effects [8]. As an ABPA therapy, amphoteric B has been investigated. Omalizumab's efficacy in treating moderate-to-severe allergic asthma has led to a surge in interest in and use of ABPA, a steroid-sparing drug, with almost universal reports of decreased steroid needs [9,10].

The benefits of treating ABPA discovered through routine testing in asthma patients have not been established. The long-term prospects for ABPA are uncertain. However, early disease detection and therapy suggestions result in favorable outcomes. Patients who are not treated eventually get respiratory failure and irreversible lung fibrosis.

## Conclusions

Our case highlights that patients with bronchial asthma may develop ABPA, which is a concerning finding. Although ABPA in bronchial asthma is not common, it should be included among differential diagnoses of non-resolving persistent respiratory infection in patients with bronchial asthma. Early diagnosis and management are mandatory to prevent the long-term morbidity associated with irreversible lung changes and mortality.
